# Granular cell tumor of the brain: case report and review of literature

**DOI:** 10.1093/jscr/rjad701

**Published:** 2023-12-30

**Authors:** Shyam Duvuru, Vivek Sanker, Deepak Pandit, Sheezah Khan, Sara Alebrahim, Tirth Dave

**Affiliations:** Department of Neurosurgery, Apollo Specialty Hospitals, Tamil Nadu, India; Team Erevnites, Trivandrum, India; Team Erevnites, Trivandrum, India; Department of Neurosurgery, Trivandrum Medical College Hospital, Trivandrum, India; Team Erevnites, Trivandrum, India; Yerevan State Medical University, Armenia; Team Erevnites, Trivandrum, India; Yerevan State Medical University, Armenia; Team Erevnites, Trivandrum, India; Faculty of Medicine, Mansoura University, MMPME; Team Erevnites, Trivandrum, India; Bukovinian State Medical University, Chernivtsi, Ukraine

**Keywords:** granular cell tumors, Schwann cells, malignant giant cell, astrocytoma

## Abstract

Granular cell tumors are rare tumors that develop from Schwann cells, which are glial cells surrounding neurons of the peripheral nervous system, which serve in the process of myelination. Granular cell tumors are rarely associated with the central nervous system in humans. In this report, we analyze a patient with granular cell tumor and review the current literature.

## Introduction

Granular cell tumors (GCTs) are soft tissue tumors [1], originating from Schwann cells, thereby possessing characteristic pathological findings. These can behave as either benign or malignant, affecting the gastrointestinal tract, skin, and oral cavity more frequently. However, granular cell tumors may occur in any part of the body [2]. They often are classified as benign, but it has been discovered that <2% of the total cases of granular cell tumors can be malignant [3, 4]. Certain genetic disorders, including Noonan syndrome and neurofibromatosis, may include GCTs as a clinical finding [5]. There are numerous types of GCTs, including diffusely infiltrative pattern granular cell tumor, epithelioid appearance granular cell tumor, spindle cell appearance Abrikossoff’s tumor, granular cell myoblastoma, granular cell nerve sheath tumor, granular cell schwannoma, and much more [6, 7]. Definitive management of GCTs is surgical resection [8]. These are usually small (typically 1–3 cm), painless, solitary, and can be asymptomatic initially in case of benign lesions. GCTs are slow growing and tend to have a good prognosis if diagnosed early in malignant lesions, whereas benign does not require any typical course of action.

Granular cell astrocytoma (GCA) is a rare type of malignant brain tumor and is usually positive for glial fibrillary acidic protein (GFAP), S100, CD68, and epithelial membrane antigen [9]. GCTs of the brain tend to be malignant, more frequent in females, and can remain asymptomatic until detected by a biopsy. GCAs are made up of massive astrocytic cells with plenty of eosinophilic granular cytoplasm, which react with the GFAP and S100 proteins. Except for the GCA, most GCTs of the central nervous system (CNS) and other body parts tend to be benign; yet, when malignant, these tumors are rare and often difficult to treat [10]. Generally, all GCAs occur in the cerebral hemispheres and are a type of infiltrative brain tumor, with most reported cases occurring in the suprasellar region. GCAs can be seen, although very rarely infratentorial within the cerebellum or spinal cord [11].

## Case report

A 39-year-old male presented to the outpatient department with decreased word output, inability to complete sentences, and difficulty in reading. He had occasional headaches and vomiting. There were no episodes of seizures and no history of weakness of limbs. He did not have any other comorbidities and no similar history in the family.

**Figure 1:**
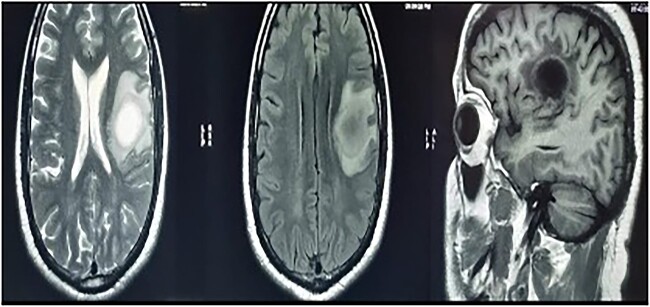
MRI brain with contrast—a heterogeneous irregular rim of an enhancing lesion with a size of 3.6 × 3.4 × 2.4 cm appearing in the left frontal subcortical region, with mild perilesional edema extending into the corona radiata and external capsule.

MRI brain with contrast revealed a heterogeneous irregular rim of an enhancing lesion with a size of 3.6 × 3.4 × 2.4 cm appearing in the left frontal subcortical region, with mild perilesional edema extending into the corona radiata and external capsule (Fig. 1). Due to an increased intracranial pressure, there was a mild midline shift of 5.3 mm toward the right side. Moreover, there was no evidence of intralesional hemorrhage or calcification seen.

**Figure 2:**
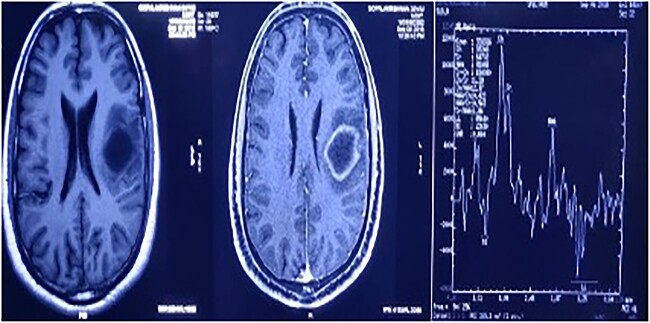
MRS showed an increased choline, reduced NAA, and prominent lipid/lactate peaks; (Ch/NAA-4.0) (Ch/Cr-1.7).

A magnetic resonance spectroscopy (MRS) was performed and revealed an increased choline, considered as a marker of neoplasms, reduced NAA, and prominent lipid/lactate peaks. (Ch/NAA-4.0) (Ch/Cr-1.7) (Fig. 2).

**Figure 3:**
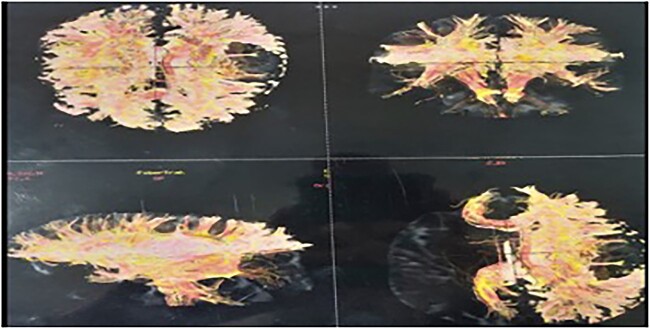
Tractography showing destruction of the left superior longitudinal fasciculus, superior occipito-frontal fasciculus, and partial destruction of the left cortical spinal tract.

A tractography was also performed, showing destruction of the left superior longitudinal fasciculus, superior occipito-frontal fasciculus, and partial destruction of the left cortical spinal tract (Fig. 3). Gray and white matter differentiation was maintained and no abnormal signals were visualized in the rest of the cerebral hemispheres. There was subtle contrast enhancement.

**Figure 4:**
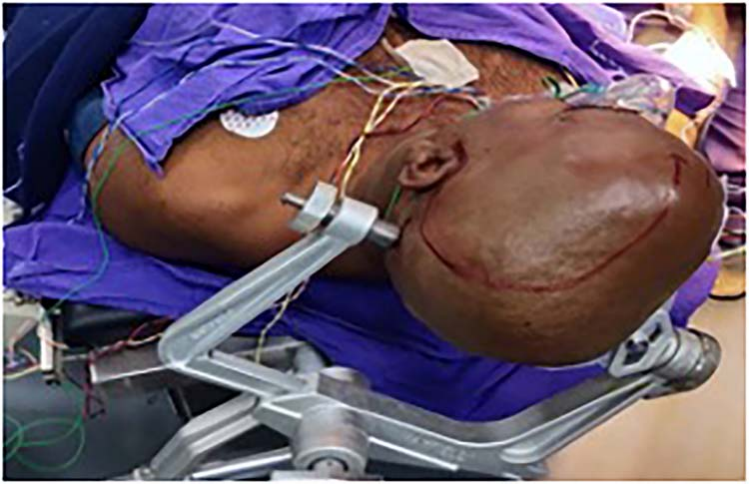
The patient was positioned in the supine position, with the head fixed with a three-pin holder.

**Figure 5:**
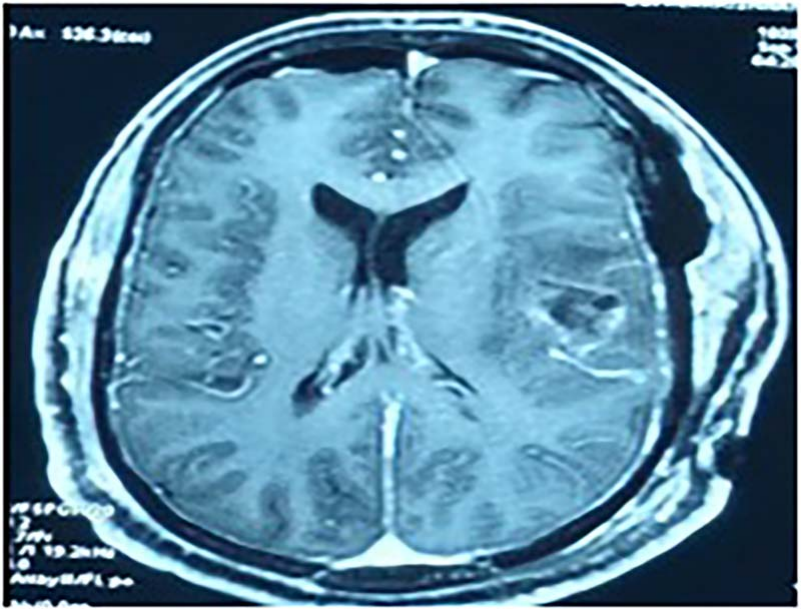
Contrast MRI depicting the tumor cavity and gross total resection.

In view of the location of the lesion and the involvement of the speech area, an awake craniotomy was planned for resection of the lesion. The goal of the surgery was maximal safe resection with function preservation. The patient was positioned in the supine position, with the patient’s head fixed with a three-pin holder (Fig. 4), and ultrasound was used to localize the lesion before opening the dura. The lesion was identified and microsurgical gross total resection (Fig. 5) was completed by using speech mapping techniques. Post-procedure, the patient had intact speech and had no new onset deficits.

**Figure 6:**
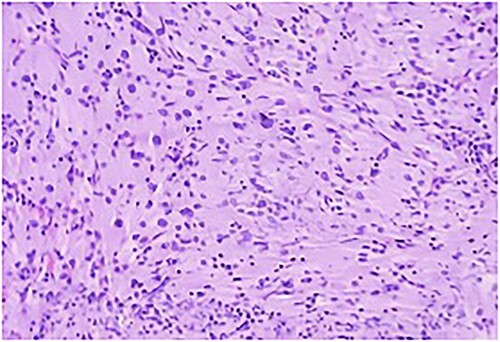
Section study shows a poorly defined mass composed of sheets of cells separated by thin collagenous bands; cells are round to polygonal with abundant eosinophilic cytoplasm with coarse granules; cell border is distinct at places with a syncytial pattern at some area; nuclei is large and vesicular.

The histopathology (Fig. 6) was reported as granular cell tumor. Immunohistochemistry also confirmed the same. Considering the nature of the lesion, the tumor board decided to give radiation to the tumor bed along with chemotherapy and follow-up imaging. The patient has completed radiotherapy and is tumor free at 4 years follow-up, without any deficits.

## Discussion

Granular cell tumor was determined to be originating from peripheral nerves due to its similar resemblance to peripheral nerves and to Schwann cells, which was shown by immunohistochemistry and electron microscopy [12]. According to their location, GCTs may have different cell sources and noticeably varied clinical symptoms. GCTs have been observed in a number of CNS regions. GCA, also known as cerebral GCT, is an uncommon variant of infiltrative astrocytoma that contains a significant number of granular cells. There have only been around 70 cases of GCA documented in the literature [10]. But unlike GCTs, which are often benign, GCAs frequently present as an aggressive glioblastoma. The cerebellum, pineal gland, and spinal cord have all been reported in a very small number of cases, but the cerebral hemispheres account for the majority of cases. Most hemispheres involve more than one lobe, with the parietal lobe appearing most frequently, followed by the frontal, temporal, and occipital lobes in that order [13, 14]. Patients with GCA are often between the age range of 27 and 83 (median age = 58 years). The male to female ratio among patients is 2:1, with a male predominance [12].

From a histological perspective, macrophages and granular tumor cells might be mistaken. Because of this, it is critical to distinguish GCA from a variety of reactive lesions, including multiple sclerosis, progressive multifocal leukoencephalopathy, and cerebral infarction [15]. In addition to considerable nuclear enlargement and atypia, GCAs frequently exhibit extensive eosinophilic nucleoli, characteristics that are not common in reactive CNS lesions in macrophages. To differentiate GCA from other disorders, it is also useful to look for a conventional infiltrating astrocytoma component and tumor cells that are bigger than macrophages [16].

Histologically, GCAs with WHO Grades II and III might display aggressive behavior. Tumors of Grade 2 were those that occasionally exhibited nuclear atypia, but lacked mitosis. But the presence of an uncommon mitosis does not transform the tumor into an anaplastic astrocytoma. Tumors classified as Grade 3 have hypercellularity, evident nuclear atypia, and noticeable mitotic activity [15]. The combination of conventional infiltrating astrocytoma and high-grade neoplastic findings in histology supports the diagnosis of GCA. Granular cell tumor histology demonstrates strong GFAP immunostaining, and its association with traditional astrocytoma suggests an astrocytic origin [10].

High-grade astrocytoma overlaps with genetic anomalies found in GCAs, which are not always specific. These include epidermal growth factor receptor amplification, homozygous deletion of CDKN2A, allelic losses at Loci 1p, 8p, 9p, 10q, 13q, 17p, 19q, and 22q, as well as methylation of the MGMT promoter [13]. Nearly all patients, including those with low-grade lesions, had loss of 9p and 10q. Several high-grade GCA cancers had TP53 mutations as well as simultaneous deletions of p14 and p16. Despite being a frequent occurrence in high-grade astrocytomas, epidermal growth factor receptor amplification was not seen in a small group of GCA [15].

Due to the aggressive nature of GCAs, most patients die within the first year of diagnosis. Patients exhibit symptoms such as new onset seizures, headache, nausea, impaired vision, disorientation, aphasia, and hemiparesis. Patients with GCAs were treated surgically and then postoperatively followed by chemotherapy and radiotherapy [14].

A few similar cases in the literature are described below (Table 1).

**Table 1:** List of similar published articles.

Study; year	Demographic features	Past history	Duration of illness	Investigations	Treatment given
A case of recurrent granular cell tumor; 2007 [8]	16-year-old female	Previous history of granular cell tumor	3-year history of a painless mass on the right side of her neck	Clinical examination: ~2 × 1 cm of a yellowish, solid, nodular mass was seen. Ultrasonography: to rule out metastases, cervical lymph nodes were examined.	Under local anesthetic, the lesion was removed. Postoperative treatment: silicone blocks and topical steroid therapy were used. Follow up.
Granular cell tumors in the CNS; 2016 [10]	29-year-old female	Amenorrhea	12 months	MRI	Surgical: total resection, pterional approach.
Granular cell tumors in the CNS; 2016 [10]	44-year-old male		None	MRI	Surgical: subtotal resection, trans-sphenoidal approach.
Granular cell tumors in the CNS; 2016 [10]	50-year-old female	Bilateral visual deficits	3 years	MRI	Surgical: total resection, pterional approach.
Granular cell tumors in the CNS; 2016 [10]	9-year-old male	Lumbodorsal pain	2 months	MRI	Surgical: subtotal resection, laminectomy.
Granular cell tumors in the CNS; 2016 [10]	12-year-old female		None	MRI	Surgical: subtotal resection, laminectomy.
Granular cell tumors in the CNS; 2016 [10]	25-year-old female	Epilepsy	3 weeks	MRI	Surgical: total resection, left frontal craniotomy.
Granular cell tumors in the CNS; 2016 [10]	45-year-old female	Facial hypesthesia	2 years	MRI	Surgical: biopsy, transnasal approach.
Granular cell tumors in the CNS; 2016 [10]	29-year-old male	Occipital pain	1 year	MRI	Surgical: subtotal resection, far lateral craniotomy.
GCA of the pineal region; 2015 [11]	16-year-old male	Parinaud syndrome, raised intracranial tension	1 month	MRI: 3 × 3 × 4–cm iso-intensity lesion and homogeneous contrast enhancement	Surgical: subtotal resection, subtemporal approach, CSF shunt.
Granular cell variant meningioma; 2019 [12]	49-year-old female	Unsteady walking	None	MRI: sized at 5.3 × 6.7 × 5.2 cm and situated in the right parietal and temporal area.	Surgical: total resection, craniotomy
GCA; 2018 [13]	81-year-old female	Seizure and left-sided hemiparesis	None	CT: hypodensity in the right temporal lobe; MRI: subcortical white matter mass in the right temporal occipital region.	Surgical: total resection
Granular cell tumor of the neurohypophysis; 2018 [14]	28-year-old male	Factor-VII deficiency	2 years	Clinical examination: decrease facial and body hair; ophthalmological examination: bilateral optic atrophy; MRI: big suprasellar mass compressing pituitary and optic chiasm.	First: trans-sphenoidal endoscopy Second: surgical subtotal resection; transcranial approach.
GCA; 2012 [16]	75-year-old male	8-year history of hypertension and MI 5 years back	3 months	CT and MRI: 6.5 cm mass seen in the frontal lobe and anterior genu of corpus callosum; Biopsy	

## Conclusion

Granular cell tumors of the brain are extremely rare lesions. Majority of the GCTs in the CNS tend to be benign and only <2% of the tumors detected are malignant, referred as GCA. Almost all GCAs are found in the cerebral hemispheres having distinct morphologic features. Unfortunately, malignant granular cell tumors are associated with aggressive clinical behavior, often difficult to treat, and require expertise and precision.

## Data Availability

The data used to support the findings of this study are restricted by the ETHICS BOARD in order to protect PATIENT PRIVACY. Data are available from the corresponding author for researchers who meet the criteria for access to confidential data.
